# Optimization of Polyolefin-Bonded Hydroxyapatite Graphite for Sustainable Industrial Applications

**DOI:** 10.3390/polym15061505

**Published:** 2023-03-17

**Authors:** Ahmed A. Bakhsh

**Affiliations:** Department of Industrial Engineering, Faculty of Engineering, King Abdulaziz University, Jeddah 21589, Saudi Arabia; aabakhsh@kau.edu.sa

**Keywords:** polyolefins, mechanical, thermal, nano fillers, industrial rubber, hydroxy-apatite, exfoliated graphite, electrical characteristics

## Abstract

As a means of introducing environmental responsibility to industrial applications, the usage of biobased composite materials has been encouraged in recent years. Polymer nanocomposites utilize polyolefins increasingly as a matrix, owing to the diversity in their features and prospective applications, even though typical polyester blend materials, such as glass and composite materials, have garnered greater attention from researchers. The mineral hydroxy-apatite, or Ca_10_(PO_4_)_6_(OH)_2_, is the primary structural component of bone and tooth enamel. Increased bone density and strength result from this procedure. As a result, nanohms are fabricated from eggshells into rods with very tiny particle sizes. Although there have been many papers written on the benefits of HA-loaded polyolefins, the reinforcing effect of HA at low loadings has not yet been taken into account. The purpose of this work was to examine the mechanical and thermal characteristics of polyolefin-HA nanocomposites. These nanocomposites were built out of HDPE and LDPE (LDPE). As an extension of this work, we investigated what would happen when HA is added to LDPE composites at concentrations as high as 40% by weight. Carbonaceous fillers, including graphene, carbon nanotubes, carbon fibers, and exfoliated graphite, all play significant roles in nanotechnology owing to the extraordinary enhancements in their thermal, electrical, mechanical, and chemical properties. The purpose of this study was to examine the effects of adding a layered filler, such as exfoliated graphite (EG), to microwave zones that might have real-world applications for their mechanical, thermal, and electrical characteristics. Mechanical and thermal properties were significantly enhanced by the incorporation of HA, notwithstanding a minor decrease in these attributes at a loading of 40% HA by weight. A higher load-bearing capability of LLDPE matrices suggests their potential usage in biological contexts.

## 1. Introduction

Applications for standard polymers have become more diverse as a result of polymer blends and grafts. Polymer blends include a wide range of products that combine two or more polymer components into a single blend or network [1]. Several types of materials may be described by just one term. It is not only conceivable but also extremely usual to combine two polymers into a single material, to improve the qualities that each polymer gives on its own. Many polymer blends exhibit phase separation, with the extent of phase separation depending on the specific mix. Depending on the composition, glass transition temperature, and phase continuity, these multiphase component polymer systems [2] may provide a broad spectrum of qualities, from toughened elastomers to high-impact plastics.

### Polymer Blends

Polymer blends, which have similar qualities to metal alloys, are created when at least two different polymers are mixed. There are now many different kinds of polymeric materials that can be produced by combining different kinds of polymers. Although no new monomers or chemical processes [3] have been discovered, this has not stopped a revolution in the study of materials science. Combining existing polymers with a known characteristic set of commercialized qualities not satisfied by any of them alone offers the benefit of a new scale of research and development with minimal expenditure, as opposed to producing new monomers and polymers to generate a comparable property profile. This is because combining existing polymers with a confirmed set of desirable qualities [4] that cannot be achieved by any one of them alone opens up new research and development scales. Lower costs associated with expanding production and bringing a product to market are potential advantages. When compared to single polymers, blends of various polymers may occasionally offer a more appealing combination of property profiles. Unique monomer/polymer blends, on the other hand, are completely novel.

The macroscopic and microscopic morphology is formed, in part, by the thermodynamic and rheological characteristics [5] of the constituents and the techniques of compatibilization. The “macro morphology” of a polymer mix describes the size and structure of the macromolecular phases that occur. These two variables may be used to define the phenomena that occur during compounding or mixing. Due to their low entropy of mixing, most polymer mixes are incompatible with one another.

Whether or not two polymers may be combined without precipitation is determined by the free energy of mixing, which includes both entropic and enthalpic components [6].

(1) $$\Delta G_{mx}\;=\Delta H_{mu}-T\Delta S_{mx}\;=\Delta E_{ma}+P\Delta V_{mx}-T\Delta S_{wx}\;$$

When at least two different polymers are combined to create a new material with varying properties, polymer blends are formed, which are similar to metal alloys. The blending of polymers has allowed for the synthesis of a plethora of one-of-a-kind polymeric materials, therefore, revolutionizing the area of materials science, without necessitating the discovery of a new monomer or the development of a new chemical procedure. The ability to combine existing polymers with proven characteristics that set the commercialized properties [7] not met by any of them singularly offers the advantage of a new scale of research and development at marginal expense, as compared to the cost of developing new monomers and polymers to yield a similar property profile. The fact that these polymers go together so well makes this a real possibility. During both the expansion phase and the transformation phase into a commercial enterprise, the low cost of financing is beneficial. In many cases, it is not possible to achieve the desired balance of properties in a composition by using a single polymer or monomer, but a blend of polymers may be able to do so. This is because numerous different monomers combine to form a polymer mix.

The macroscopic and microscopic morphologies are affected by the thermodynamic and rheological characteristics [8] of the components as well as the compatibilization procedures. In polymer blends, the “macro morphology” describes the size and form of the macromolecular phases. The formation of macroscopic phases during compounding or blending is referred to as the “macro morphology” of polymer blends. As the entropy of mixing most polymer combinations is so low, it is not practical to use them together.

Whether or not two polymers can be combined depends on their free energy of mixing [9]. Therefore, there might be both entropic and enthalpic energy:

(2) $$\frac{\Delta G_{mat}}{RT}=\left(\frac{\phi_{A}}{N_{A}}\right)ln\phi_{A}+\left(\frac{\phi_{G}}{N_{B}}\right)ln\phi_{B}+\chi_{FN}\phi_{A}\phi_{B}$$

When two or more types of polymers are combined to form a new material with unique characteristics, a blend of polymers is an inevitable consequence. The resultant material has properties similar to metal alloys. The ability to blend polymers has allowed for the production of several novel polymeric materials, sparking a revolution in materials research. There was no need to find a new monomer or develop a new chemical process to achieve this. Combining existing polymers with a proven characteristic set to commercialize properties not met by any of them singularly offers the advantage of new scales of research and development at marginal expense, in comparison to the cost of developing new monomers and polymers to yield a similar property profile. This is a real possibility when existing polymers are combined with a well-established set of desirable characteristics that none can provide on their own. When starting business and expanding existing operations, low expenditure is beneficial. If the objective is to generate a more acceptable balance of qualities, a composition consisting of a single polymer or monomer might be better than a composition consisting of a mix of polymers.

The thermodynamic and rheological properties of the components and the compatibilization procedures influence both the macroscopic and microscopic morphologies [10]. A polymer blend’s “macro morphology” characterizes the shape and size of its macromolecular phases. The creation of macroscopic phases during the compounding or blending process is referred to as “macro morphology” [11]. Unfortunately, most polymer mixtures cannot be employed together because of their poor mixing entropy.

To ascertain whether or not two polymers may be mixed, the free mixing energy [12] is used. Entropic and enthalpic energy is detectable in this system. The necessary condition for phase separations to occur is

(3) $${\left(\frac{\partial^{2}\Delta G_{a}}{\partial \phi_{2}^{2}}\right)}_{T,\beta }=0$$

where Gm is Gibb’s mixing free energy. If it is established that the miscibility parameter Gm is zero, we obtain the mixing configuration entropy, Sn0. Specifically, an interaction of Mer 1 and Mer 2 is the only way to produce a uniform mixture [13]. These interactions may result in ionic or dipolar interactions. In general, polymer miscibility decreases with increasing temperature but improves with increasing pressure.

For mixtures to be miscible by boiling, the mixture quality must be stated.

(4) $$P=P_{l}\Phi_{1}+P_{2}\Phi_{2}+l\phi_{1}\Phi_{2}$$

When two or more polymers are combined, the resulting material may be tailored to specific applications [14]. Producing metal alloys is similar to this procedure. Many novel polymeric materials may be created by the mixing of polymers, which has transformed the area of materials science without the requirement to discover a new monomer or new chemistry. Combining existing polymers with a proven characteristic set of commercialized properties not met by any of them singularly offers the benefit of a new scale of research and development at marginal expense, as opposed to the process of developing new monomers and polymers to produce a similar property profile. This differs from the standard practice of creating new types of monomers and polymers. The ability to grow operations with little initial investment is a major advantage. A composition consisting of a mixture of polymers, as opposed to a composition consisting of a single polymer or monomer, may be preferable to attain a more acceptable balance of properties.

The thermodynamic and rheological characteristics of the constituents, in addition to the compatibilization processes, affect the macroscopic and microscopic morphologies. Macro morphology describes the size and shape of the macromolecular phases formed in polymer blends during the compounding or blending processes. Due to their low entropy in mixing, the vast majority of polymer mixtures are useless when combined.

The free mixing energy [15] is used as a criterion to establish whether two polymers are compatible with one another. Both entropy and enthalpy may be thought of as kinds of energy that are present in the system.

## 2. Background Study

Yoshihiko Ohama conducted research into syntactic foam material model assessment [16]. They carried out their analysis by employing the RVE method that is provided by commercial FE software (ANSYS) and placing balloons at random. They created an inclusion microstructure that does not overlap and has six distinct volume fractions—0.1, 0.2, 0.3, 0.4, 0.5, and 0.6, respectively. They assessed both the viable Youthful’s modulus and the powerful Poisson’s proportion by exposing the RVE to occasional uniaxial limit conditions. This was so they could look at the RVE. They contrasted the analytical model with the FE models in order to precisely predict the effective elastic module of syntactic foams.

Utilizing Laguerre tessellation models, Michele T. Byrne and Yurii K. Gun’ko investigated the effect that cell wall thickness and size have on the strength of closed-cell foam [17]. In 2017, their study was published. In order to allow it to withstand both compressive and shear loads, the RVE model received two hard shells—one at the top and one at the bottom. The top shell was given a constant velocity in the downward and sideways directions for the compressive and shear loadings, respectively, while the bottom shell was held stationary. Using a commercial FE software package known as ABAQUS/Explicit, they were able to successfully resolve the issue in both modes. They discovered that as the diversity of cell size and cell wall thicknesses increased, so did the compressive and shear strengths. Additionally, they discovered that compressive strength was more susceptible to variation than shear strength.

Utilizing the RVE method with a void percentage of forty percent, Eric J. H. Chen and Benjamin S. Hsiao investigated the effect that particle clustering has on the tensile characteristics and failure processes of syntactic foams [18]. The numbers 0.2, 0.4, 0.6, and 0.8 stand for the four distinct levels of clustering they used. The extreme values of =0 and =1 are regarded as 39, respectively, indicating that the particles are fully grouped and evenly distributed. They published a stress–strain curve that was a homogenized response of RVE with five different clustering levels. At first, the response was linear elastic in a particular strain range, and then it became nonlinear. They reported this to us. Additionally, they demonstrated that as the degree of particle clustering rises, syntactic foams’ tensile strength and fracture strain gradually decrease. They discovered that this was the situation. They stated that the simulation curves’ nonlinear parts were not as smooth as the experimental curve, and they also reported the experimental stress–strain curve for comparison with the simulated results. Utilizing an RVE model with few particles is the direct cause of these significant reductions in stiffness.

A dynamic finite element method for modeling blow molding and thermoforming was developed using the Mooney–Rivlin hyperplastic material model [19]. The degree of freedom of the parison remained fixed on the solid boundary until the simulation’s conclusion because they assumed that the contact between the parison and the mound was sticky. They used a unique lumping method to put the explicit central difference time integration scheme into action. Discretization was carried out for triangular components. Instances of blow shaping and thermoforming of convoluted objects, such as a container with a handle and modern box math, were utilized to show the newfound interaction.

Ref. [20] used an extrusion blow molding method to study the particle formation. To understand the thickness distribution of the blown part, they used the FE software program ANSYS Polyfold to simulate blow-molded components and then compared the results of the simulation to those of the experiments. Their examination depended on a similar investigation. They looked at the comparison between the amount of blowing agent and the speed of extrusion and found that increasing the amount of blowing agent increases the comparison’s length and perimeter, while increasing the speed of extrusion makes the comparison’s length slightly shorter. The blow molding simulation was carried out at blowing pressures of 0.3 and 0.7 MPa because the mound is not symmetrical. It was found that the container’s thickness does not stay the same around its perimeter, regardless of the pressure used. The portion that was blown at 0.7 MPa had a surface quality that was extremely regular, homogeneous, smooth, and brilliant, despite having a thickness that was slightly lower than that of the portion that was blown at 0.3 MPa. Shahzad et al.’s study, which was published in 2015, utilized experimental data as an input into the FE software tool to carry out an analysis that resulted in the establishment of the hyperplastic material model (ABAQUS). In order to collect the necessary input data for the identification of the material model, they carried out tests with a total of four distinct loadings—uniaxial tensile, volumetric, planar, and biaxial—in order to collect the data. The coefficients of the models were retrieved so that they could be simulated using the curve fitting tool that is included in ABAQUS. After running simulations and comparing the results to the test data, they came to the conclusion that the Yeoh model was the best option.

### Research Motivation

For polymers such as polypropylene (PP) that have crystalline structures and low melt strengths, the production of olefin-based foams using a variety of processing processes has proven to be a problematic subject for both academic researchers and industrial manufacturers. Even though researchers have developed and examined a wide variety of foams created by a variety of processes, and although these foams are routinely used in industries, there are still unknown areas of study that may be further expounded upon. In addition to these significant advancements, further developments in terms of both experimental and modeling studies are required for better understanding and control of process parameters, particularly on processing conditions, such as pressure drop rate, temperature, the content of blowing agent in a matrix, etc., which govern the nucleation, cell growth, cell size, structures, and cell coalescence. These conditions include pressure drop rate, temperature, the content of the blowing agent in a matrix, etc. The structure of the foam and the cell shape are both determined by the process parameters.

Yet, it has been established that the addition of a tiny number of nanoparticles may affect the structure of foams and increase their capabilities, hence, making it possible for foams to be used in applications that were not possible before. The manufacture of polymeric foams, which regulates the governing characteristics of the foams, still faces considerable problems, however. In addition, there is a pressing need for a deeper comprehension of the structure–property link in conjunction with the operating conditions. The creation of cellular structures on both the micro- and the nanoscales has been the focus of a significant amount of study and technical progress over the last several decades. Despite the advances in research and technology, there is still a deficiency in the simulation analysis of foam structure and the optimal processing condition of foam products, particularly in foam blow molding. This is especially true of the case in foam blow molding. Despite this, the technology of foam blow molding is still relatively new. While it has begun to make its way into the market, it is still in the early stages of development. The simulation analysis of these foams is an excellent tool for optimizing both the products and the process. It is feasible, via the use of mathematical models, to obtain a good agreement between the outcome of a simulation and the solution of a real-world situation. Yet, due to the intricate nature of the foam’s structure and the nature of its two-phase system, it is difficult to imitate the foam. In addition, there is a scarcity of data and mathematical models, which further complicates the task of research in this area.

## 3. Materials and Methods

Table 1 lists the many different possible combinations of saturation temperature, pressure, and other process parameters that may be used to perform PIF foaming. According to Table 1, when the PIF temperature rises, the relative density of the foams decreases and their porosity rises. Relative density decreases in a way that is directly proportional to the increase in porosity and expansion ratio.

To have a better understanding of how temperature-induced foaming works, we compiled a summary of the relevant processing parameters in Table 1. The saturation time in the TIF technique was much longer than in the PIF method when the materials were solid and the temperature was kept at room temperature. Further, the water in the tub was hotter than the PIF. The “immersion time” refers to the length of time the solid samples spent in the hot glycerol bath before turning into foam. The relative density and porosity of these foams are also shown in the table, showing that the relative density of the final foams did not differ much over the range of TIF conditions. The data support this claim since it is included in the table. The next several sections will explain why there is so little fluctuation in the relative density of TIF foams and what effect this has on the final product. Table 2 indicates the temperature, saturation pressure, saturation time and their destiny involved in industrial rubber.

Polypropylene (PP), which is a versatile polymer, has seen a rapid increase in its use as a result of its excellent performance as well as improvements in its production costs. The commercial market has shown a significant amount of interest in PP and PE blends. The addition of PE to PP has the potential to improve the material’s resistance to impact at low temperatures. Table 3 shows the properties and values of the material’s normalized mechanical.

Weld lines are an inevitable part of the manufacturing process for most big injection-molded items, such as appliances, home wares, furniture, sports goods, toys, packaging, chemical processing equipment, and industrial components. Automobiles are a good illustration of this category. In the presence of weld lines, optimizing processing parameters and determining the optimal number of modifiers are crucial for achieving the best possible features. There is no way to ensure that PE and PP work together. In terms of the materials, PP/HOPE blends do not have the best standing with regard to mechanical characteristics. Their final mechanical compatibilizations are lacking in comparison to the compatibilization of the individual parts.

If included, this component would act as a computerizing agent in the amorphous regions of both polymer species. PPIPE mixes’ compatibility may be enhanced in a variety of ways, one of which is by adding the copolymer EPR.

The reaction’s aftereffects include the breakdown of PP and the branching or cross-linking of poly(ethylene). The process has to be controlled with a high degree of precision to be optimized. Increased radiation or reactive compatibilization may decrease the mixture’s crystallinity. To satisfy the need for thermoplastic materials with the characteristics of vulcanized rubber, rubber-toughened PPIHDPE mixes are essential. According to [21], when HOPE serves as the matrix, PP lamella migrates into the dispersed phase of EDPM, whereas in the case of HDPE, PE lamellae migrate into the PP matrix. PPIHDPE/EPDM refers to tertiary mixes of polypropylene, ethylene, and ethylene propylene. The PP crystallization was unaffected by the addition of PE. As a pair, PP and PE produce results that are in direct opposition to one another. A two-phase structure is produced by the combination of two different polymers. When the mix is put under extreme stress, its structure might compromise its performance. Dispersion and thermal and mechanical degradation rates may be enhanced by increasing the mixing duration or intensity. Excessive stretching causes incompatibility between the PP and PE, which degrades their mechanical qualities. Evidence suggests that the ethylene-propylene copolymer provides stronger adhesion than the separate monomers provide to one another.

The adhesion between the polymers is the most influential element in determining the ductility-related properties of immiscible blends. The adhesion between ductile and brittle parts might be improved to increase ductility. The Flore-Huggins interaction parameter, *a*, defines the extent to which individual segments diffuse over the interface between components.

(5) $$a=c/{\left(\chi_{A\underline{\sigma }}\right)}^{m}$$

(6) $$\rho_{f}=\frac{a}{a-b}\rho_{water\;}$$

where it is the density of the foam sample, *a* is the apparent mass of the sample in air, and *b* is the apparent mass of the sample completely immersed in water.

(7) $$R_{\rho }=\frac{\rho_{f}}{\rho_{s}}$$

The relative density *R* is defined as the ratio of density of foam to the density of unfamed solid (*ρ_s_*):

(8) $$R_{v}=\frac{1}{R_{\rho }}$$

They produce brittle incompatible combinations when melted together and injected into molds, which is a process known as melt mixing. Because of the poor ductility of the material, injection molding an immiscible mix produces a weld line that is perpendicular to the direction in which the load is applied.

Numerous investigations have been conducted, and the results of those studies have uncovered a variety of elements that lead to the weaker weld lines of homopolymers [20,21,22]. Quantifying the contents of each bin allowed for the calculation of this size distribution. Using this size distribution as a guide, we were able to calculate the micrograph-based average cell diameter, denoted by the symbol *D_v_*:

(9) $$D_{v}={\left[\frac{\sum_{t-1}^{n}d_{i}^{3}}{n}\right]}^{1/3}$$

The cell density, also known as *N_f_*, is the number of cells that can be found in a given volume of foam. It can be calculated as follows: where *n* represents the total number of cells and *d_i_* represents the perimeter equivalent diameter of each counted cell:

(10) $$N_{f}={\left[\frac{nM^{2}}{A}\right]}^{3/2}\times R_{v}$$

The number of cells that may be found in a certain volume of foam is referred to as the cell density, and it is also denoted by the symbol *N_f_*. It is possible to compute it as follows: where *n* is the total number of cells and di is the perimeter equivalent diameter of each counted cell. This may be carried out by using the formula.

(11) $$K_{1C}=\delta {\left(\frac{E}{H}\right)}^{0.5}\frac{P}{C^{1.5}}$$

The process of injection molding is distinguished by the introduction of a complicated and nonisothermal flow into a chamber that has been sealed and cooled. An anisotropic skin core structure is produced as a consequence of this procedure in most cases. Fountain flow is used to accomplish the task of filling the mold cavity. During the molding process, the polymer that is in touch with the cooled mold wall instantly freezes, forming the skin at the location where the shear will be at its greatest.

When obtaining a material’s hardness and Young’s modulus using instrumented nanoindentation, the Oliver and Pharr (O-P) model, which is the approach that is used the majority of the time, states that the hardness (*H*) is stated as the Oliver and Pharr (O-P) model:

(12) $$H=P_{mad}/A_{ct}$$

where *P_mad_* represents the maximum load that is applied and $A_{ct}$ represents the actual contact area that exists between the indenter and the material. In Oliver and Pharr’s work, it is stated that the polynomial form of $A_{ct}$ may be represented as:

(13) $$A_{ct}=24.56\;h_{e}{\;}^{2}+C_{1}\;h_{c}+C_{2}\;h_{e}{\;}^{1/2}+C_{3}\;h_{e}{\;}^{1/4}+\cdots \cdots +C_{8}\;h_{e}{\;}^{1/128}\;$$

where *C_1_* through *C_8_* are constants that may be determined with the help of a standard calibration procedure, and he is the penetration depth, which can be determined with the help of the formula that is provided below:

(14) $$h_{c}=h_{max}-k\left(P_{mad}/S\right)$$

where *k* is less than 0.75 in the case of a Berkovich indenter. Another way to express the contact stiffness (*S*), also known as the slope of the load versus depth of penetration plot shown in Equation (15), is as follows:

(15) $$S={\left(dP/dh\right)}_{h=max}=aC_{A}E^{*}\sqrt{A_{cr}}$$

where is 1.034, *C_A_* equals 2/, and E* is the effective Young’s modulus for a Berkovich indenter. Following the O-P paradigm, E* may be expressed as:

(16) $$1/E^{*}=\left(1-v_{i}^{2}\right)/E_{i}+\left(1-v_{s}^{2}\right)/E_{s}$$

where the subscripts *i* and (*s*) denote the indenter *i* and sample *s*, respectively, and where (*E*) and (*v*) represent the Young’s modulus and the Poisson’s ratio, respectively. For a Berkovich indenter, the *E_i_* and *v_i_* pressures are commonly calibrated to be 1140 GPa each.

**Figure 1:**
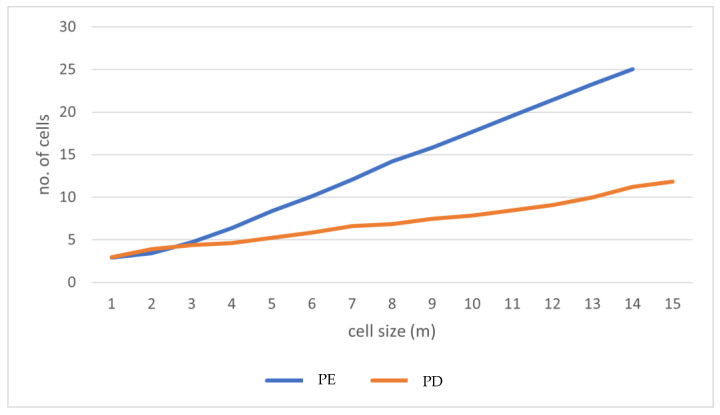
Cell size vs. number of cells.

**Figure 2:**
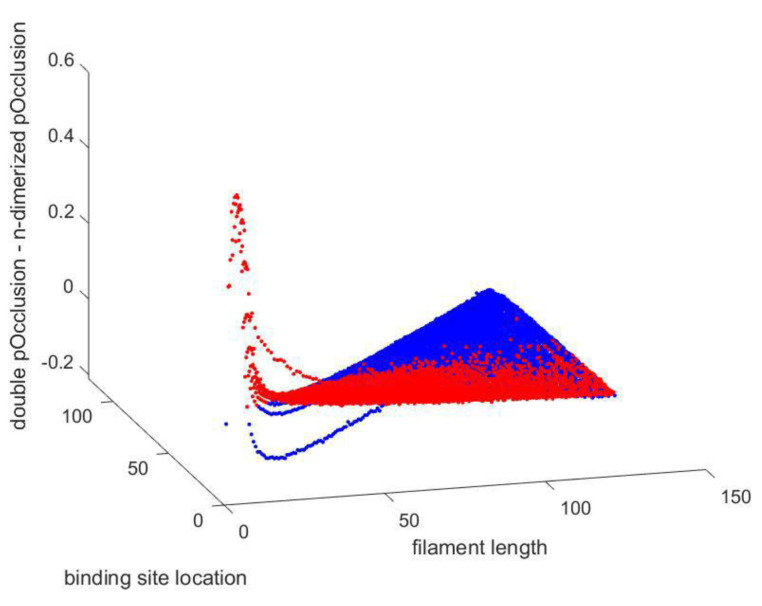
Variation in torque with time of HDPE-HA composites during mixing.

Particles that are flexible and scattered will have their shape stretched in the direction of the flow as the flow moves through them. The agitated core will experience less stress as a result, and it will have more time to cool down. The result of this labor-intensive process is a skin-core structure with two layers. There have been reports that PP/EPDM and HDPE/PA-6 blends have poor weld line strength due to the form of the skin cores of the blends’ respective polymers. The influence of processing variables and compatibilizers on the behavior of HOPE and PP blends along the weld line. To create test specimens with and without weld lines, a semi-automatic plunger-type injection molding machine was used. The weld line coefficient (WLC) is the value that is derived by comparing the characteristics of the sample that contains the weld line to those of the sample that does not include the weld line [22]. This occurs when two samples are prepared under identical conditions. The temperature at which the material is processed has an effect on the yield stress as well as the elongation at break, as shown in Figure 1 and Figure 2. Increasing the processing temperature of HDPE from 190 °C to 250 °C results in an improvement in the material’s WLC.

The rate of cooling is determined by several variables, one of which is the thermal diffusivity of the melt.

## 4. Results

All of the composites were made using a twin-roll mixer with rolls of 45 cm by 15 cm in diameter (a calendar) for ten minutes at a maximum temperature that remained constant at 140 °C. Following the melting of the polymer matrix for one minute, the fillers and additives were then combined and added to the slurry. After the mixing process, composites were recovered with a thickness of 1.5 mm.

The specific gravity of a substance is determined by comparing the mass of that substance to the mass of the same volume of deionized water at a temperature of 23 °C. The specimen is first measured and recorded while it is suspended in air, and then again when it is held submerged in distilled water at a temperature of 23 °C using a sinker and a wire. For each formulation, the density was determined using the standard ASTM D792 and then compared to the value that had been computed. This was so that we could be certain that the formulation had been correctly put together.

At room temperature, the tensile characteristics were evaluated using a Tinius Olsen H10KT dynamometer with an elongation speed of 250 mm/min. The length after stretching was 20 mm 0.5 mm, while the breadth and thickness of the specimens were, respectively, 3.0 mm and 2.0 mm 0.2 mm (according to the standard ISO 37 type 2).

Following the requirements of the international standard ISO 1133:1, the flow parameters were evaluated using an instrument called a Melt Flow Index (MFI).

The FEI Quanta 450 ESEM FEG (FELMI-ZFE, Graz, Austria) was used for the scanning electron microscopy (SEM) analysis that was performed so that the particle morphology of the fillers could be determined.

A laser diffraction technique was used to determine the particle size distribution of the fillers at the D50 level. For these studies, a Rasterizer 2000 manufactured by Malvern Analytical (Malvern, UK) was used.

An Olympus BTX 470 II diffractometer (Olympus, Shinjuku, Tokyo) was used to carry out an X-ray powder diffraction (XRD) analysis.

TA Instruments’ TGA Q500 (New Castle, DE, USA) was used during all of the thermogravimetric tests that took place. Samples ranging from 10–15 mg were put in crucibles made of Al_2_O_3_, and the runs were performed in high-purity nitrogen. Over a range of 50–1000 °C, the rate of heating was 20 °C per minute.

All of the flame tests and LOI measurements were carried out using ASTM D2863 by using an SA ASSOCIATES Oxygen Index instrument on specimens with dimensions of 10 mm by 6 mm by 3 mm. A burner flame was applied to the top of a bar that was positioned vertically in a test column that runs with a combination of oxygen and nitrogen. The LOI value is the minimum percentage of oxygen (%) that must be present in the gas mixture to sustain the combustion of the item being measured. The starting concentration of oxygen is decided in a completely arbitrary manner.

HOPE grade 50 MA 180 from Indian Petrochemicals Limited (IPCL, Mumbai, India) and pp Repoll H 200 MA from Reliance Petrochemicals (Ahmedabad, India) were used in the mixing and molding process. A Thermos Hake Rheochord 600 mixer (Thermo Fisher Scientific Inc., Waltham, MA, USA) with a 69 ern’ roller-type rotor chamber was also utilized in this process. Heredia Unmiters’ EPDM grade 301 T, TMQ antioxidant, and dicotyl peroxide were used. After being combined in the mixing chamber, the substance was immediately transferred to a two-roll mill to have its particle size decrease.

### 4.1. Determine the Effect of Melt Temperature on the Strength of PP and HDPE Weld Lines

The weld line strength of semicrystalline materials, such as PP and HDPE, is impacted not only by the rate of crystallization but also by the amorphous sections that remain in the solidified samples. WLC, on the other hand, decreases as the temperature is elevated, most likely because processes that are detrimental to WLC begin to take place.

In terms of PP, the WLC for yield stress and elongation at break starts to dramatically fall at around 240 °C. This is the temperature at which this change occurs. Given that tertiary hydrogen enhances PP’s vulnerability to degradation, this was, to some extent, to be expected. The values of the material’s normalized mechanical properties are shown in Table 3. HDPE has a very high elongation at break, whereas PP has a much lower value. Fractures in PP do not exhibit necking, and PP samples collected from weld lines exhibit a high degree of brittleness.

The cell size distributions that were generated from the micrographs and depicted in Figure 1 gave further validation of this behavior when they were examined more closely. Although there is substantial variety in cell sizes, the size distributions shown in Figure 1 demonstrate a clear and gradual shift to the right (bigger cell sizes) as the temperature rises. This occurs even though there is broad variation in cell sizes. This pattern is in agreement with the prediction that PIF cells would continue to an expansion in size as long as the temperature is increased, at least up to 170 °C.

According to these distributions, the average bubble size for PIF at each of the three temperatures is as follows: 3 m when the temperature is 150 °C (PPF-01), 4 m when the temperature is 160 °C (PPF-02), and 312 m when the temperature is 170 °C (PPF-03). The dramatic expansion of the bubbles that occurred when the temperature was raised from 160 to 170 °C may have been caused by a reduction in the viscosity of the polymer that occurred with an increase in temperature. This reduction in the viscosity of the polymer resulted in a lower barrier to the development of cells. Cells may also burst as a result of a drop in viscosity brought on by an increase in temperature, which is another potential cause of this phenomenon.

**Figure 3:**
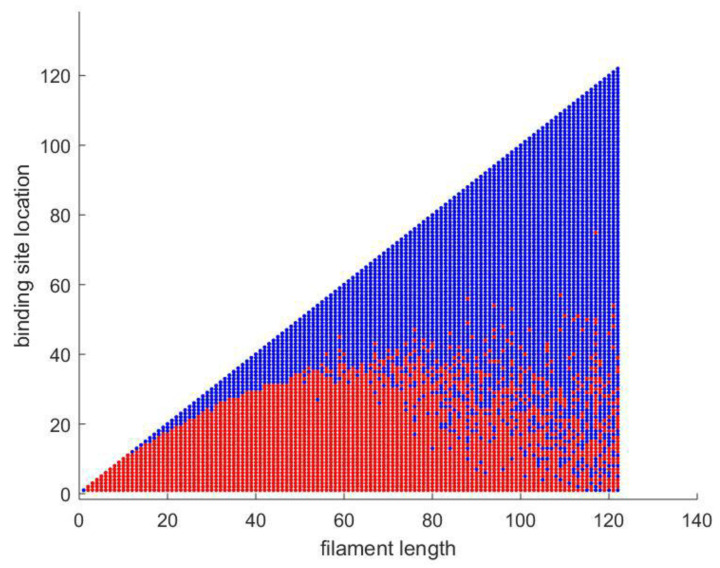
Variation in MFI of HDPE-HA composites with HA loading.

At a temperature of 180 °C, the average cell diameter was measured to be 115 m. (PPF-04). Further examination of the morphology of the PIF-made foam at 180 °C, as shown in Figure 3, reveals a significant gradient in the cell sizes, which range from the edge to the center of the foam. The cells on the edge are smaller in size in comparison to the cells in the middle. Because the foam morphology exhibits a significant departure at 180 °C, which is in contrast to the trend seen between 150 and 170 °C, it is possible to argue that PIF has a limiting temperature condition at 180 °C for the pressures that are used. This is because the trend seen between 150 and 170 °C can be attributed to the fact that the foam expands as the temperature rises. In addition, at temperatures higher than 180 °C, the solubility of carbon dioxide in the polypropylene matrix begins to noticeably decrease, while the diffusivity out of the polypropylene matrix begins to increase. This results in poor sorption and retention of carbon dioxide at the pressure that was used in this investigation. It is possible that the amplification of these effects at such temperatures is to blame for the lack of foam generation in specimens that were subjected to PIF temperatures higher than 180 °C.

### 4.2. Torque Studies

As illustrated in Figure 2 the time-dependent torque variations that take place during the melting process of pure HDPE and HDPE-HA composites, respectively. In the illustration, the notation Hex refers to HDPE that has had an unspecified quantity of HA added to it. The torque rises as more polymer is injected, and then decreases while melting takes place, then stabilizing after around two minutes have passed. At this point, HA is added, and the mixture is agitated for a full ten minutes before being poured into the container. The torque is maintained at the same level during the whole mixing procedure, indicating a sufficient amount of filler dispersion across the matrix following the parameters described.

### 4.3. Melt Flow Index

Figure 3 illustrates how the amount of HA loading affects the pace at which the melt flows. MFI exhibits a minor decline when HA loading was stopped, but only slightly. This demonstrates that the inclusion of HA results in a decrease, although a minor one, in the flow characteristics. The melt flow index is a measurement of the entanglements in the polymer matrix, which may be caused by either chemical or physical cross-links. Since HA causes a small increase in the number of entanglements in the polymer chain, this leads to a decrease in the MFI.

### 4.4. Tensile Properties of HDPE-HA Composites

**Figure 4:**
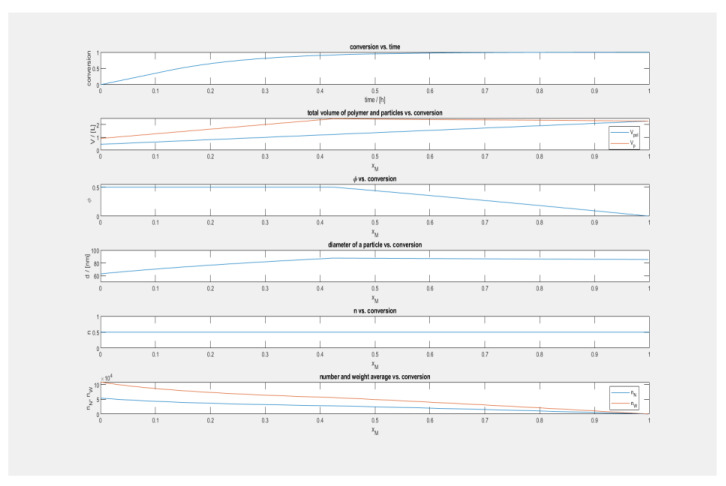
Stress–strain curves of HDPE-HA composites.

Figure 4 illustrates the stress–strain curves of both plain HDPE and composite materials. The elastic area of pure HDPE may be identified by its distinct yield point, which is followed by neck propagation and culminates in strain hardening. After it has been yielded, it can sustain extensive extension, which results in a long plastic area. This occurs as a direct consequence of the entanglements, which cause the molecular chains to lengthen. When HA was added to the mix, the structural properties of the stress–strain curve remained unchanged up to a loading of 1.5 weight %. After this, there is a discernible decrease in the plastic area that takes place. When the weight % of HA reaches three, the necking process fails without any strain hardening taking place. The loading of one weight % of HA is necessary to achieve the maximal amount of elongation. The capacity of the evenly scattered filler particles to perform the function of fracture stoppers is responsible for the improved ductility of the material. As a result, sufficient time is given the opportunity to pass. The tensile strength shows a small rise with HA loading, reaching its maximum value at a loading percentage of one weight percent. Further, also increasing with HA loading is the tensile modulus, which reaches the highest value of 32.7% in the HD1H composite.

### 4.5. Compressive Properties

**Figure 5:**
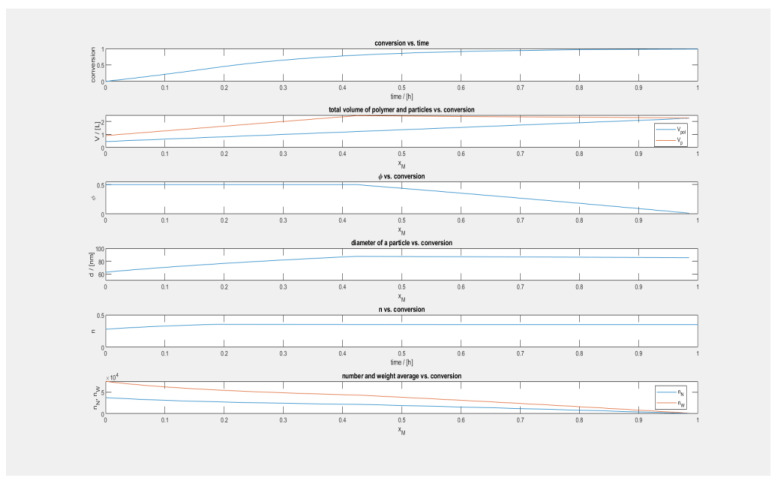
Variation in compressive modulus of HDPE-HA composites with HA loading.

According to the findings shown in Figure 5, the compressive modulus increases with increasing HA loading up to a maximum of a 54.8% increase for HD1.5H. After reaching this peak, the rise almost levels out. The ability of the filler particles to close cracks and flaws that are perpendicular to the applied load is responsible for the increased compressive strength that is brought about by the addition of fillers. The increased compressive modulus is an indication of the increased load-bearing capacity of the material.

### 4.6. Flexural Properties

**Figure 6:**
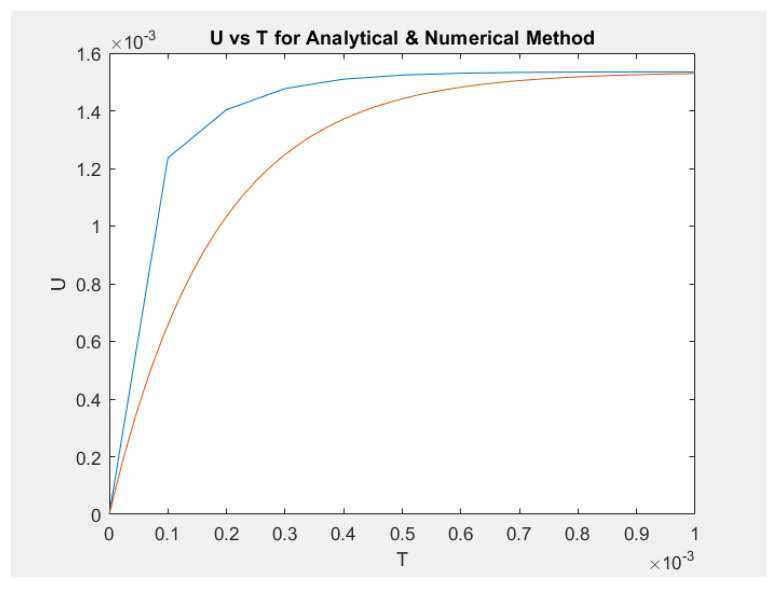
Variation in flexural strength.

The increased flexural property of the HDPE-HA composites is evident in Figure 6. Both flexural strength and modulus increased with increased HA content, substantiating the enhanced resistance toward bending forces. This is contributed by the stiffening effect of the HA particles.

### 4.7. Impact Strength

**Figure 7:**
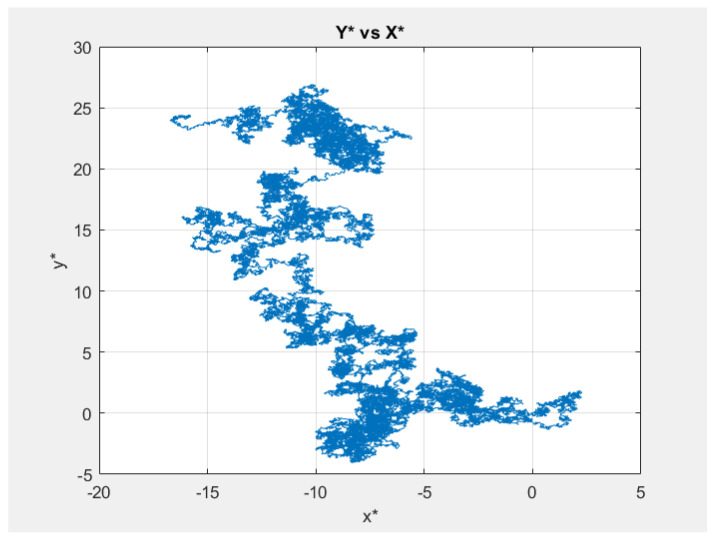
Variation in impact absorbance energy of HDPE-HA composites.

The effect of HA on the impact absorbance energy is obvious from Figure 7. Impact properties increase with increased HA content. The well-dispersed HA particles help to transfer stress effectively during the impact test.

### 4.8. Dynamic Mechanical Analysis

**Figure 8:**
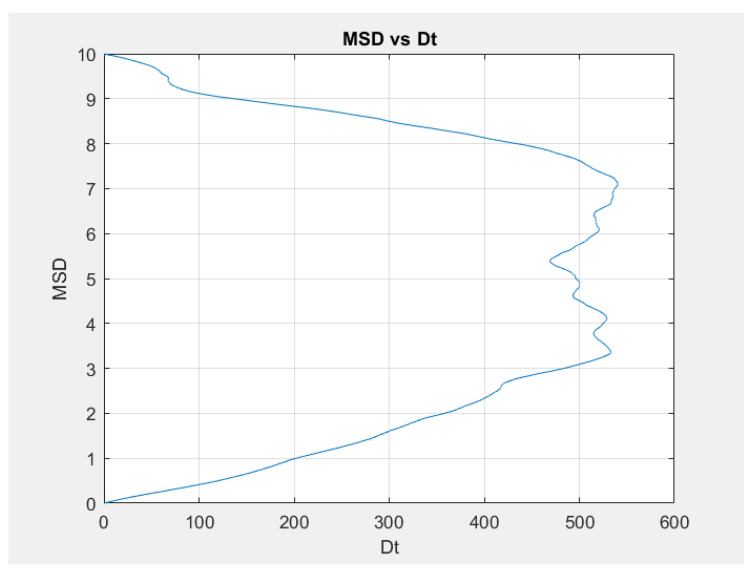
A comparison of the dynamic mechanical characteristics of pure HDPE and HDPE-HA composites.

A comparison of the dynamic mechanical characteristics of pure HDPE and HDPE-HA composites is shown in Figure 8. When the temperature is increased, there is a detectable decrease in the storage modulus across all of the samples. This finding lends credence to the hypothesis that the increased temperature is responsible for the increased mobility of polymer chains. Both storage modulus and loss modulus grow with increasing HA concentration; however, the values for HD1H’s storage modulus and loss modulus are the greatest. This is evidence that the micro-HA rods and the polymer matrix are in close contact with one another and are transmitting stress to one another. It may be deduced from the fact that there is practically no movement in the tan delta peak that HA has very little to no effect on damping characteristics. When the amount of EG loading was increased, it was found that all of the nanocomposites’ dielectric permittivity, dielectric loss, alternating current (AC) conductivity, and absorption coefficient all increased. The incorporation of conductive EG causes electromagnetic waves to be attenuated before they have the opportunity to enter the material, which results in a reduced depth of the material’s skin. The dielectric heating co-efficient in polyolefin-EG composites experiences a significant decrease proportional to the increasing quantity of EG present in the material. When the frequency of the heating is increased, the depth of the skin and the heating coefficient both decrease as shown in Table 4.

Table 5 presents analysis of proposed work. It is indicated that composites made of high-density polyethylene (HDPE) and ethylene glycol (EG) demonstrated the largest improvement in dielectric properties when compared to composites made of low-density polyethylene (LLDPE) and polypropylene (PP)-EG, respectively. We were able to introduce HA into LLDPE at loadings of 10, 20, 30, and 40 weight percent with the use of melt mixing, and we then examined the impact of the HA at each of these different loadings. The inclusion of HA led to a notable improvement in the mechanical and thermal characteristics of the material as a whole, despite the fact that there was minor degradation in the properties when the loading was increased to 40% HA by weight. The higher load-bearing capability of the LLDPE matrix implies that it may be utilized in biomedical applications.

Extensive biological testing led to this result. Melt mixing was used to create LLDPE-HA-EG hybrid composites, taking advantage of the synergy between the various fillers in the polymer matrix. Because of the enhanced properties provided by hybrid composites, LLDPE, which was previously solely usable for packaging, containers, and toys, may now be put to a far larger variety of uses. We evaluated the “in vitro” and “in vivo” possibilities of high HA-loaded polyolefin-HA composites and polyol-fin-HA-EG hybrid composites for orthopedic applications and ratio of their effects is shown in Table 6.

The materials’ structures were characterized, and their crystallinity levels were evaluated, using wide-angle, two-angle x-ray microscopy (WAXM) from a range of 4 to 800. In addition to the HDPE peaks at 2 = 21.70, 24.10, 30.20, and 36.40, which correspond to reflections in the (100), (200), (210), and (020) planes, respectively, in all three samples and LLDPE, one can also observe the HA peaks at 2 = 25.90, 31.90 (highest-intensity peak), 39.90, 39.7, 46.80, and 49.50. Peak 260, which is distinctive of HA, is blended with the peak of HA, making the two peaks difficult to identify from one another.

**Figure 9:**
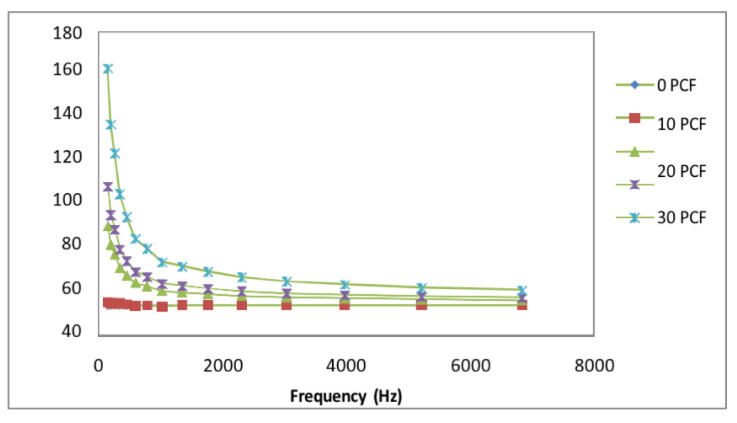
Variation in dielectric constant of PTH-coated fiber/PE composites with frequency.

Figure 9 defines the composites’ dielectric constant drops with frequency, and the effect is particularly prominent at low frequencies. As the electric field is changed, the dipoles’ orientations also change. Lower-frequency dipoles are capable of maintaining phases with the field’s oscillation. Dipole orientation and polarization will lag behind the frequency rise, and this is represented by a decrease in the dielectric constant.

### 4.9. Protection against Electromagnetic Interference

**Figure 10:**
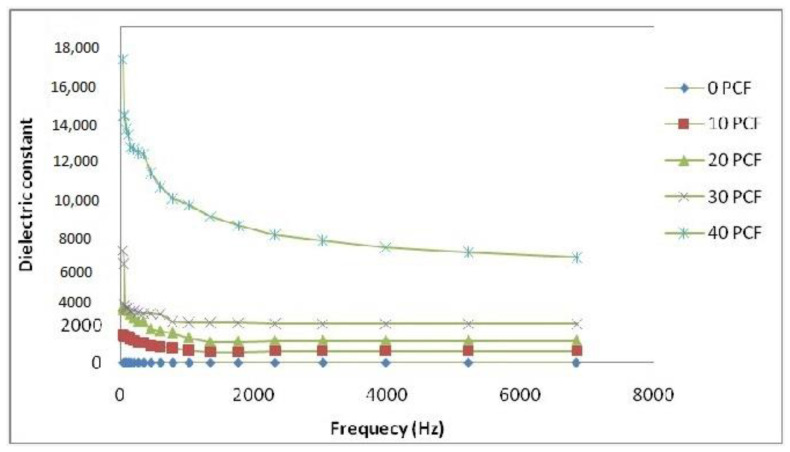
Variation in dielectric loss of HDPE-coated fiber/PE composites with frequency.

Figure 10 explain the variation with regard to the impact of HDPE-coated fiber content and frequency, dielectric loss also mimics the dielectric constant. With each cycle of the alternating electric field, the composites’ dipoles swap positions. The friction between the dipole and the surrounding molecules causes the substance to heat up. The dielectric loss of composites may be traced back to this unavoidable thermal energy loss. Increases in fiber content led to an increase in dielectric loss and heat loss due to an increase in the number of dipoles. Lowered frictional heat dissipation manifests as decreased dielectric loss as the frequency at which the dipoles lag behind the alternating field rises.

**Figure 11:**
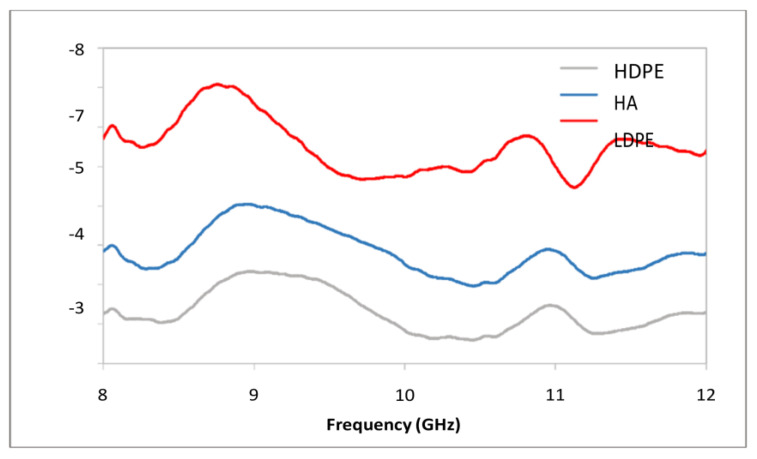
Total EMSE of PCF/PE composites in X band.

Figure 11 displays the total EMSE of PCF/PE composites, while Figure 11 displays the EMSE caused by absorption. Inferences may be made about the primary mechanism of shielding, and they point to absorption, allowing reflection loss to play a minor part. Nevertheless, unlike HDPE/PE composites, PCF/PE composites have a very poor shielding effectiveness. This might be because the HDPE-coated fibers contain moisture, reducing conductivity, and/or because the coated fibers disturb the crystallites in the matrix, further reducing conductivity. For optimal shielding, sample thicknesses greater than 1 mm are being investigated. It has been shown that when the percentage of HDPE in a material rises, the frequency of the peak moves down.

#### Protection against Electromagnetic Interference

**Figure 12:**
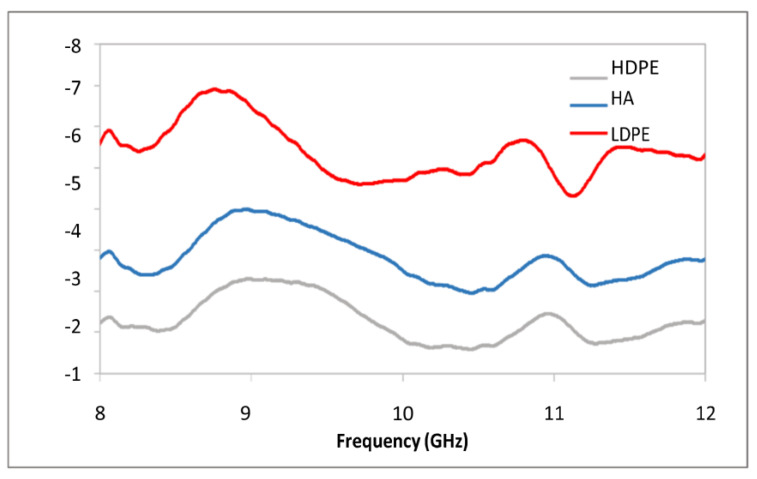
EMSE due to absorption of HDPE /PE composites in X band.

Figure 12 illustrates that Polythiophene-coated cellulosic fibers might be incorporated into a common general-purpose thermoplastic polymer, high-density polyethylene, to create conducting composites. Scanning electron microscopy reveals weak matrix-coated fiber interaction due to polar–nonpolar incompatibility. As compared to HDPE/PE composites, the mechanical qualities of PCF/PE composites are inferior. Yet, a lower modulus is beneficial in applications that need adaptability. Moisture is trapped in the coated fibers, according to thermal experiments. Matrix thermal deterioration may be postponed with the use of coated fibers. Because of the presence of moisture in the cellulosic fibers, the conductivity of the HDPE coating and, by extension, the composites is considerably lower than that of HDPE/PE composites. With the use of dynamic mechanical analysis, we can see how a network of coated cellulose fibers forms. The composites are well-suited for use as capacitors due to their high dielectric constant and minimal dielectric loss. Due to their poorer conductivity, PCF/PE composites provide less protection from electromagnetic interference than HDPE/PE composites.

## 5. Conclusions

The research concluded that the mechanical properties of PP were enhanced by the addition of HA. Composites loaded with 1% HA showed the greatest increase in mechanical parameters, including tensile strength, flexural strength, and compressive modulus.

It would appear that public concern regarding the mechanical response of biocomposite materials is preventing their widespread use and production in industries, particularly in the primary load-carrying section. This is mainly due to the lack of technical data on characteristics, such as tension, compression, fatigue, impact, flammability, etc., on which a dependable engineering design relies significantly for the purpose of failure start and progression prediction. Research into the design and performance of industrial applications, such as product standards, conceptual breakthroughs, lab-scale concepts, durability studies, and degradation models, is crucial for their widespread use in industry. Growing the market for industrial applications might necessitate adjusting aspects, such as supplier–user relations and regulations controlling the use of nano-fillers, in industrial applications utilized in the packaging of food.

Increasing commercialization of these materials is anticipated as the public’s awareness of environmental issues grows, as more cost-effective manufacturing methods become widely accessible and as new applications for these materials are identified. Commercialization has been delayed where these resources are most plentiful—in developing nations—because of a lack of adequate research activities. Researchers are challenged with addressing challenges connected to norms and product standards for these components, notwithstanding the renewability and recyclability of industrial applications. Industrial applications developed for structural usage should conform to regulations governing the disposal of massive garbage loads.

Both the scientific and business communities have a keen interest in industrial applications materials, but it has been challenging to find suitable substitutes for synthetic composites in this area. They would be a difficult replacement for conventional synthetic composites in industrial applications due to their low mechanical and thermal properties. Accomplishing the goal of high-performance industrial applications will necessitate careful consideration of the following factors: identification of raw material, extraction procedure, sustainable crop development, application of industrial applications interfacial qualities, material processing and product production, safe service life, and product design. Notwithstanding the potential benefits of multi-fiber reinforcements and mixes of diverse polymers, it seems that they have been the subject of very few studies. More study should be devoted to this area because of the interesting possibilities for new uses. The failure mechanisms caused by the thermo-mechanical–chemical processes of industrial applications, as well as the effects of environmental ageing, need further study. In order to be widely used, materials for industrial applications need to perform as expected, last as long as expected, be reliable, and be easily maintained.

Both the storage and loss moduli improved when HA was put into place. The thermal stability of the composites was improved by the addition of HA. Among these fillers, graphite stands out due to its usefulness as a nano-filler in the form of graphene layers or nano-scale layered stacks. These nanoscale stacked layers have the chemical properties of CNTs and the structural properties of layered silicates. This has the potential to greatly enhance the composites’ conducting capabilities in addition to their mechanical and thermal properties.

## Figures and Tables

**Table 1:** Pressure induced in industrial rubber.

Pressure Induced Foaming					
Sample	Saturation Pressure (MPa)	Saturation Temperature (°C)	Saturation Time (h)	Relative Density	Porosity
PP-00	−	−	−	1.0	−
PPF-01	7	150	2	0.431±0.048	0.569
PPF-02	7	160	2	0.347±0.050	0.653
PPF-03	7	170	2	0.205±0.009	0.795
PPF-04	7	180	2	0.207±0.039	0.793

**Table 2:** Temperature induced in industrial rubber.

Temperature Induced Foaming					
Sample	Saturation Pressure (*MPa*)	Saturation Time (*h*)	Glycerol Temperature (°C)	Relative Density	Porosity
PPF-05	7	24	180	0.473±0.017	0.527
PPF-06	7	24	190	0.512±0.038	0.488
PPF-07	7	24	200	0.509 ± 0.026	0.491
PPF-08	7	24	210	0.518±0.017	0.482

**Table 3:** Properties of HDPE.

Properties	Values	Test Methods
At 192C and 1.98 kg	0.06	ISO1132
Input density	921	ISO1181
Input tensile		ASTM 881
Elasticity behavior	1150	
Thermal range	110	
Processing range	210–240	ASTM 227

**Table 4:** Concentration of proposed work.

Sample	Concentration *μ* bol.cm ^−2^	
	by UV	by Temperature
HDPE	0.92	0.9
LLDPE	0.42	0.4
HA	1.08	1.0

**Table 5:** TGA analysis of proposed work.

Sample	Temperature for Weight Loss		Peak Max Ma	Residue at 600 °C (%)	
	10%	25%			
HDPE	426.1	443.8	461.4	474.1	0.08
LLDPE	453.1	477.9	495.2	492.7	38.9
HA	473.2	490.8	505.3	500.6	40.6

**Table 6:** Effect of ratio.

N	Effect of Ratio	α
1	715.813	3.7603
2	−369.475	3.9685
3	33,311.620	−3.4034

## Data Availability

Not applicable.
